# Nexilin, a Cardiomyopathy-Associated F-Actin Binding Protein, Binds and Regulates IRS1 Signaling in Skeletal Muscle Cells

**DOI:** 10.1371/journal.pone.0055634

**Published:** 2013-01-30

**Authors:** Andrew Lee, Fumihiko Hakuno, Paul Northcott, Jeffrey E. Pessin, Maria Rozakis Adcock

**Affiliations:** 1 Department of Laboratory Medicine and Pathobiology, University of Toronto, Toronto, Ontario, Canada; 2 Department of Animal Sciences and Applied Biological Chemistry, The University of Tokyo, Tokyo, Japan; 3 Department of Medicine, Albert Einstein College of Medicine, Bronx, New York, United States of America; University of Texas Health Science Center at Houston, United States of America

## Abstract

Insulin stimulates glucose uptake through a highly organized and complex process that involves movement of the glucose transporter 4 (GLUT4) from intracellular storage sites to the plasma membrane. Previous studies in L6 skeletal muscle cells have shown that insulin-induced activation and assembly of insulin receptor substrate 1 (IRS1) and p85α the regulatory subunit of the Type 1A phosphatidylinositol-3-kinase (PI3K), within remodeled actin-rich membrane structures is critical for downstream signalling mediating the translocation of GLUT4. The mechanism for localization within actin cytoskeletal scaffolds is not known, as direct interaction of IRS1 or p85α with F-actin has not been demonstrated. Here we show that nexilin, a F-actin binding protein implicated in the pathogenesis of familial dilated cardiomyopathies, preferentially binds to IRS1 over IRS2 to influence glucose transport in skeletal muscle cells. Nexilin stably associates with IRS1 under basal conditions in L6 myotubes and this complex is disassembled by insulin. Exposure of L6 myotubes to Latrunculin B disrupts the spatial patterning of nexilin and its transient association with IRS1. Functional silencing of nexilin has no effect on insulin-stimulated IRS1 tyrosine phosphorylation, however it enhances recruitment of p85α to IRS1 resulting in increased PI-3, 4, 5-P_3_ formation, coincident with enhanced AKT activation and glucose uptake. By contrast, overexpression of nexilin inhibits transmission of IRS1 signals to AKT. Based on these findings we propose that nexilin may tether IRS1 to actin-rich structures under basal conditions, confining IRS1 signaling to specific subcellular locations in the cell. Insulin-elicited release of this constraint may enhance the efficiency of IRS1/PI3K interaction and PI-3, 4, 5-P_3_ production at localized sites. Moreover, the selective binding of nexilin to IRS1 and not IRS2 may contribute to the differential specificity of IRS isoforms in the modulation of GLUT4 trafficking in skeletal muscle cells.

## Introduction

Development of resistance to the metabolic actions of insulin on peripheral tissues such as skeletal muscle, adipose tissue and liver is recognized as an early step in the progression to type 2 diabetes mellitus. Central to the development of insulin resistance are defects in insulin-stimulated glucose uptake in skeletal muscle which accounts for ∼80% of post-prandial whole body glucose disposal [1]. It is well established that binding of insulin to its cell surface receptor, one member of the large family of receptor tyrosine kinases, induces the redistribution of the glucose transporter 4 (GLUT4) from intracellular membrane compartments to the plasma membrane where it catalyzes the uptake of glucose, a rate-limiting step for glucose metabolism [2]. Although numerous pathways have been implicated in insulin-dependent GLUT4 trafficking, few of these fulfill the criteria of specificity that would be predicted for the unique action of the hormone on glucose homeostasis. One emerging concept suggests that spatial and temporal compartmentalization of signaling intermediates may be required to ensure the fidelity and specificity of insulin signaling. There is growing evidence that supports a critical role of the cytoskeleton in compartmentalizing insulin-dependent signals and regulating GLUT4 membrane-trafficking events, although the precise functional role and cytoskeleton-regulatory mechanisms remain enigmatic [3]–[4]. For example, insulin has been reported to induce cortical F-actin remodeling in both skeletal muscle cells and adipocytes and these actin ruffles have been co-localized with insulin signaling intermediates [5]–[10]. Furthermore, pharmacological agents that disrupt or inhibit F-actin polymerization inhibit GLUT4 translocation and glucose uptake [11]–[16]. In this regard, biochemical studies have demonstrated that IRS1/PI3K complexes are preferentially activated and tyrosine phosphorylated by the insulin receptor (IR) in an intracellular low density microsome (LDM) membrane fraction [17]–[21]. Moreover, it appears that these complexes are not membrane-associated but rather anchored to an actin cytoskeleton framework, and that this state of subcellular localization is important for IRS1/PI3K dependent mitogenic and metabolic actions [21], [22]. In a search for scaffolding proteins that may provide a link between the actin cytoskeleton and localized IRS1/PI3K signaling we have identified nexilin, an F-actin binding protein which we show binds selectively to IRS1 but not to IRS2.

Nexilin is expressed specifically in human heart and skeletal muscle where it is localized at the sarcomeric Z-disc, a key structural interface between the cytoskeleton and the sarcolemma [23]. Traditionally, the Z-disc has been viewed as the unit responsible for transmitting mechanical forces generated within sarcomeres, however, recent evidence suggests that Z-discs are also critical elements involved in signaling and disease [24]. Notably, the discovery of an increasing number of novel Z-disc proteins and their role in the pathogenesis of cardiomyopathies implicates the Z-disc as a critical component in the regulation of cardiac function [24]. In this regard, loss of function mutations in nexilin have been causally linked to the pathogenesis of familial dilated (DCM) and hypertrophic (HCM) cardiomyopathies [23], [25]. Accordingly, inactivation of nexilin in zebrafish leads to the rupture of cardiac sarcomeres and heart failure, pointing to an essential role for nexilin in the maintenance of sarcomeric integrity [23]. Interestingly, the PI3K/AKT network has also been identified as a critical hub that controls Z-disc stability and contributes to the development of pathological cardiac hypertrophy [26]–[28]. Persistent activation of PI3K/AKT axis elaborated by chronic hyperinsulinemia or transgenic expression of constitutively active AKT results in excessive cardiac growth leading ultimately to heart failure [27], [28]. In this study we provide evidence for a novel role for nexilin as a component of the insulin signalling network in skeletal muscle cells where it influences the assembly of IRS1/PI3K complexes and activation of AKT leading to glucose uptake.

## Materials and Methods

### Materials

Parental L6 myoblast cells were a kind gift from Amira Klip (Toronto, Canada) [22]. Actin antibodies, Latrunculin B, dexamethasone and 3-isobutyl-1-methylxanthine were purchased from Sigma Aldrich. Jasplakinolide was purchased from Calbiochem. IRS1-preCT, IRS2, 4G10 and p85 antibodies were obtained from Upstate Millipore. AKT, S473pAKT and T308 pAKT antibodies were purchased from Cell Signalling Technologies. Cy5-conjugated donkey anti-rabbit antibodies and Cy3-conjugated goat anti-mouse antibodies were from Jackson ImmunoResearch and Rhodamine-phalloidin from Molecular Probes. Nexilin antibodies were purchased from BD Biosciences and raised in house against the CC and ABD regions. Nexilin specific small interfering RNA (siRNA) and control siRNA oligos were purchased from Qiagen.

### Cell culture, siRNA transfection, adenoviral transduction

L6 myoblasts were maintained in minimal essential medium-alpha (alpha-MEM) supplemented with 10% fetal bovine serum (FBS) in a humidified incubator containing 5%CO2 at 37C. When experimenting on myotubes, L6 cells were cultured to the stage of myotubes in alpha-MEM containing 2% FBS. Transfections of nexilin siRNA into L6 myoblasts were performed using the calcium phosphate method. Experiments were performed 72 hours post transfection. Transfection of nexilin siRNA into L6 myotubes was performed by first transfecting siRNA (100nM) into L6 myoblasts at ∼70% confluency. The next day, the media was changed to 2% alpha-MEM and changed thereafter every 24 hours. On day 5, the differentiating myotubes were transfected again with siRNA (100 nM) in 2% FBS alpha-MEM. L6 myotubes were ready for experimentation on day 8. 3T3-L1 adipocytes were transduced with adenovirus expressing Flag-tagged nexilin-IRES-GFP (Ad-Nex) or Green Fluorescent Protein (Ad-GFP) and experiments were generally started 72 hours post infection. Latrunculin B (LB) and LY294002 pretreatments were performed by diluting drugs to a final concentration of 20 µM and 50 nM respectively in serum-depleted medium for the final 20 minutes of starvation. Jasplakinolide (Jaspk) pretreatments were performed by diluting the drug to a final concentration of 2 µM in serum-depleted medium for the final 30 minutes of serum starvation. Insulin was added to serum-starved cells at the desired concentration and indicated length of time.

### Immunofluorescence microscopy

L6 myotubes in chamber slides were fixed with 3.7% formaldehyde in PBS for 10 min and permeabilized with 0.2% Triton X-100 in PBS for 15 min. Cells were then rinsed three times with PBS and blocked with normal goat serum diluted 1:20 or with 5% BSA/PBS for 30 minutes. Cells were stained with primary antibodies or rhodamine-conjugated phalloidin for 30 min. Primary antibody detection was performed with FITC- conjugated goat anti-rabbit IgG, Cy3-conjugated donkey anti-mouse or Cy5-conjugated donkey anti-rabbit. In controls, primary antibody was omitted. Samples were examined using a Zeiss Axiophot microscope (Zeiss Inc.).

### Glucose uptake

siRNA-transfected L6 myotubes were serum-starved for 4 hrs and subsequently treated with or without insulin for 20 min. Cells were washed twice with HEPES-buffered saline solution (140 mM NaCl, 20 mM HEPES, 2.5 mM MgSO4, 1 mM CaCl2, 5 mM KCl, pH 7.4) and glucose uptake was assayed by adding HEPES-buffered saline solution containing 10 µM 2-Deoxy-D-Glucose and 0.5 µCi/mL 2-deoxy-D-[^3^H]) for 5 min. Glucose uptake was terminated by washing three times with ice-cold 0.9% NaCl (w/v). Cytochalasin B (10 µM) was included in one or two wells during glucose stimulation to determine non-specific uptake. Intracellular [^3^H]-Glucose was determined by lysing the cells with 0.1 N KOH, followed by liquid scintillation counting. Total cellular protein was determined by the Bradford method. For glucose uptake in 3T3-L1 adipoyctes, cells were transduced with Ad-GFP or Ad-Nex adenoviruses and 72 hours post infection, cells were starved for 3 hrs and stimulated with 10 nmol/L insulin for 30 minutes at 37°C. Data are expressed as mean ± SEM, assessed statistically by one-way ANOVA.

## Results and Discussion

**Figure 1:**
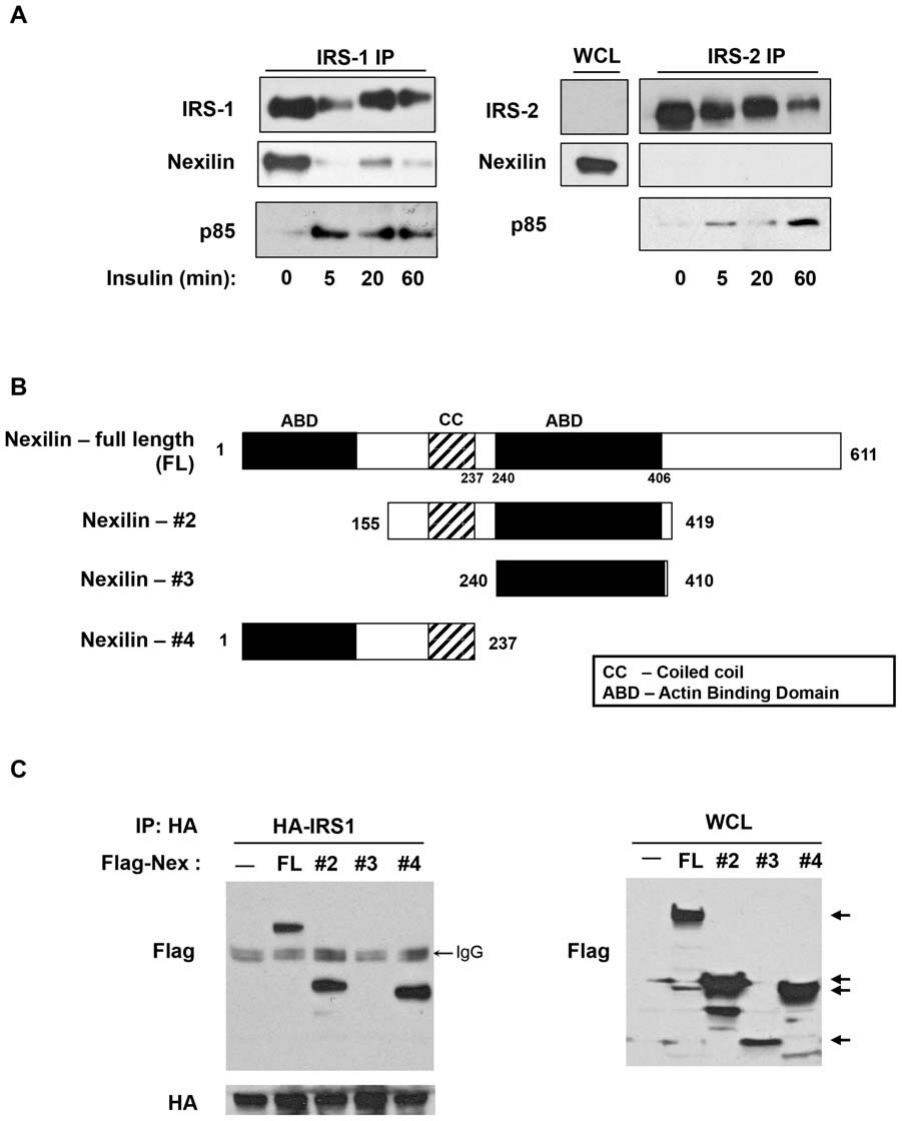
Nexilin is a novel binding partner of IRS1. *A*) Nexilin selectively binds to IRS1 in L6 skeletal muscle cells. Serum starved L6 myotubes were left untreated or stimulated with 100 nM insulin for the indicated times. Cell lysates were immunoprecipitated (IP) with either IRS1 or IRS2 antibodies (abs) and subjected to western blot analysis with the indicated abs. WCL, whole cell lysates; *B*) Schematic representation of nexilin constructs. The isolated open reading frame of s-nexilin predicts a protein of 611 amino acids (aa) and consists of a central coiled-coil (CC) domain flanked by two F-actin binding domains (ABD). Nexilin-#2 is a truncated version containing the CC and second ABD domain (aa. 155–419) Nexilin-#3 consists of the second ABD domain (aa 240–410). Nexilin-#4 contains the N-terminal ABD and CC domains (aa 1–237). *C*) HEK293 cells were transfected with either pCMV5b vector (C), full length (FL) pCMV5b/Flag-nexilin construct or Flag-tagged nexilin-#2, #3 or #4 constructs. Cells were co-transfected with HA-IRS1. Left Panel, Lysates were immunoprecipitated with HA abs and blotted with either Flag or HA abs. Right Panel, Whole cell lysates (WCL) from transfected cells were immunoblotted with Flag abs showing expression of recombinant nexilin proteins.

A proteomic search for components of the insulin signaling network in skeletal muscle cells, identified nexilin as an IRS1 interacting partner. To examine this interaction, we used L6 rat skeletal muscle cells where nexilin is abundantly expressed. IRS1 was immunoprecipitated from L6 myotubes that had been serum starved and then treated with insulin (100 nM) for 5, 20 and 60 min. Immunoprecipitated lysates resolved by SDS-PAGE showed that nexilin and IRS1 are stably associated under basal conditions, however insulin stimulation elicited dissociation of the complex coincident with recruitment of p85α to IRS1 (Fig. 1A). We next sought to determine if this interaction is specific to the IRS1 isoform. Despite the high degree of homology between IRS1 and IRS2, biochemical and metabolic studies from knockout mice and cell lines indicate that IRS1 and IRS2 do not possess redundant roles [29], [30]. For instance, whereas skeletal muscle from IRS1 deficient mice show reduced insulin-stimulated glucose transport and GLUT4 translocation [31], glucose uptake into muscles isolated from IRS2 knockout mice is unaffected [32]. Moreover, Klip and coworkers have shown that in cultured L6 cells, glucose uptake is only diminished in siIRS1-treated cells whereas IRS2 silencing does not translate into diminished insulin-dependent glucose uptake [29]. Immunoprecipitation assays in L6 cells using an IRS2 antibody revealed no evidence of interaction between nexilin and IRS2 under both basal and insulin-stimulated conditions (Fig. 1A). Thus, the selective binding of nexilin to IRS1 and not IRS2 may contribute to the differential specificity of IRS isoforms in transmitting insulin signals to downstream effectors.

We next sought to identify the region within nexilin that confers binding to IRS1. Nexilin contains two actin-binding domains (ABD), that flank a central coiled-coil domain (CC). The ABDs have been shown to bind to α-actin and β-actin in cardiac and skeletal muscle cells [23], [25]. We designed various Flag-tagged nexilin deletion constructs (Fig. 1B) and tested their ability to bind to ectopically expressed HA-IRS1 in 293 cell lysates. Our data indicate that the CC region of nexilin is required for nexilin/IRS1 binding (Fig. 1C).

**Figure 2:**
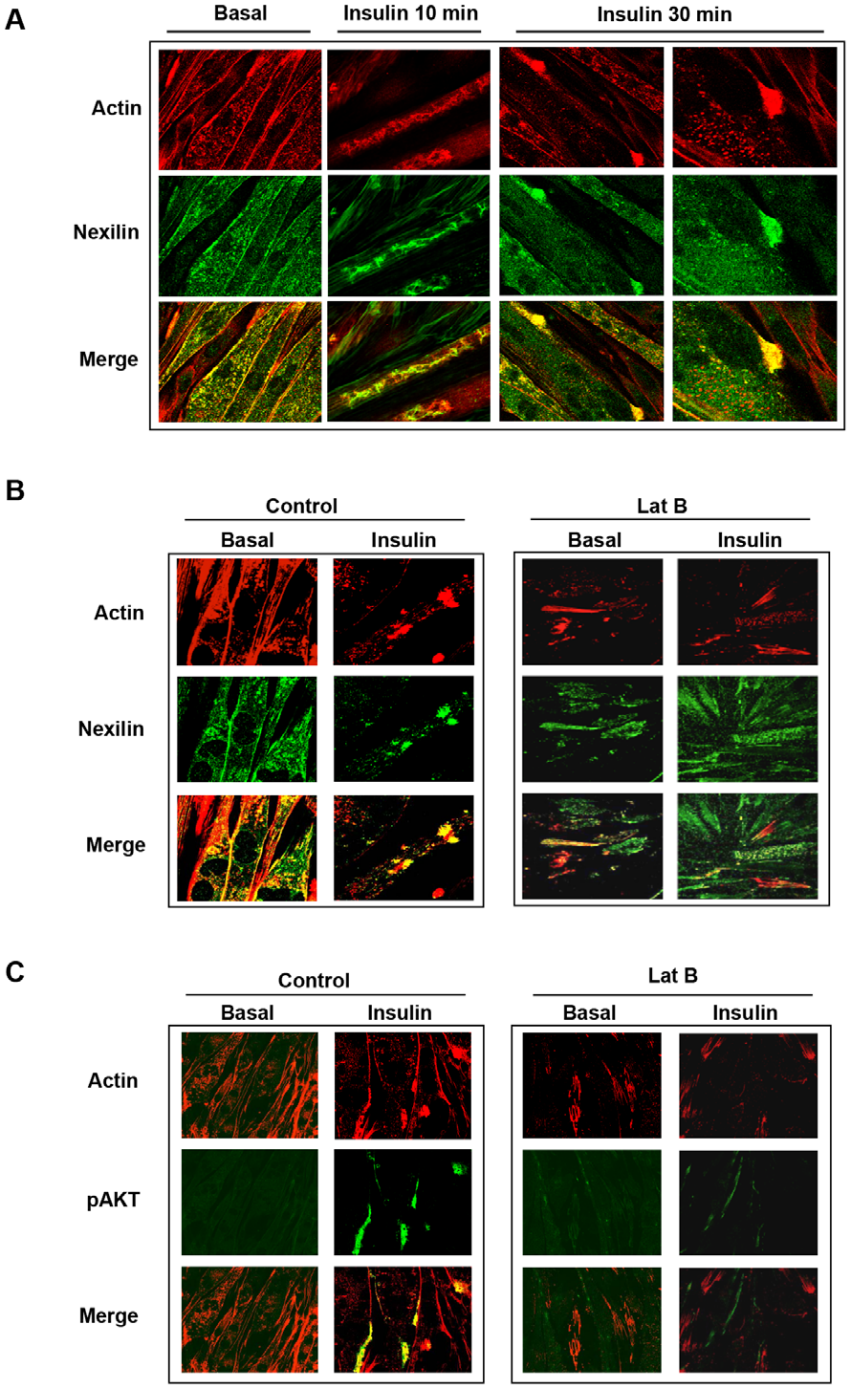
Spatial distribution of nexilin in L6 skeletal muscle cells. *A*) L6 myotubes were serum starved (basal) or stimulated with 100 nM insulin as indicated and then fixed, permeabilized and incubated with anti-nexilin abs, Cy5-conjugated secondary antibodies (green) and rhodamine-phalloidin (red). Images were obtained on a Zeiss LSM510 laser scanning confocal microscope; *B*) Serum depleted L6 myotubes were pre-incubated with or without Latrunculin B (LatB) and subsequently stimulated with 100 nM insulin for 30 minutes. Cells were stained as in *A*); *C*) L6 myotubes were treated as in *B*) and processed for visualization using phospho-Ser473 Akt abs (green) and rhodamine-phalloidin (red).

We next used immunofluorescence and confocal microscopy to determine the subcellular localization of nexilin under both basal and insulin-stimulated conditions in cultured rat L6 myotubes. In the basal state, nexilin exhibited a relatively homogeneous distribution scattered throughout the cytoplasm (Fig 2A). Following 10 min of insulin stimulation, nexilin underwent a dramatic redistribution into actin-rich membrane ruffles aligned along the longitudinal axis of the myotubes and by 30 min of insulin treatment was mobilized into distinct punctuate actin bundles at the plasma membrane. To explore whether this insulin-stimulated nexilin translocation is dependent on actin filament polymerization, we employed the drug Latrunculin B (Lat B) that scavenges actin monomers and destabilizes actin cytoskeletal organization. In these experiments, myotubes were serum starved and either left untreated or incubated with Lat B for 20 min. The cells were then incubated in the absence or presence of insulin for 30 min. As shown in Figure 2B, Lat B pretreatment prevented insulin-mediated actin remodeling and resulted in complete dispersal of nexilin. Moreover, disassembly of the actin cytoskeleton coincided with diminished Akt activation as determined by the intensity of the Ser 473 Akt phosphorylation signal (Fig. 2C). These results suggest that the spatial patterning of nexilin is linked to actin remodeling induced by insulin.

**Figure 3:**
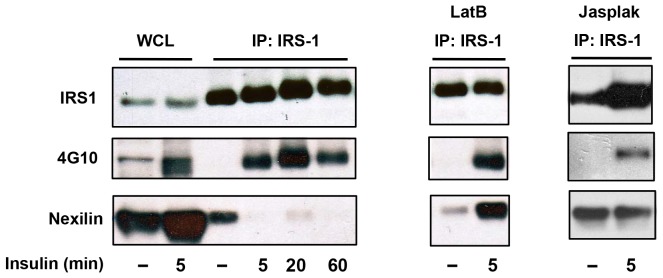
Insulin-induced dissociation of IRS1/nexilin complex is dependent on F-actin remodeling. Left panel, IRS1 was immunoprecipitated from L6 myotubes that were either starved or insulin stimulated (100 nM) for the indicated times. Immune complexes were probed with anti-phosphotyrosine 4G10 or nexilin abs. WCL, whole cell lysates; Right Panel, Latrunculin B (20 µM, 20 min) or Jaspakinolide (2 µM, 30 min) treatment of L6 myotubes is without effect on the phosphorylation status of IRS1 but inhibits insulin-induced IRS1/nexilin disassembly.

We next tested the effect of Lat B treatment on IRS1-nexilin interactions. Interestingly, while exposure of L6 myotubes to Lat B was without effect on insulin-induced IRS1 tyrosine phosphorylation, Lat B treatment blocked the disassembly of the IRS1/nexilin complex in response to insulin, suggesting that efficient dissociation of this signaling complex is dependent on dynamic reorganization of the actin network (Fig. 3). This result prompted the assessment of actin filament stabilization on IRS1-nexilin interactions. To this end, jasplakinolide, which stabilizes F-actin filaments by inhibiting filament disassembly was used to treat L6 myotubes at the end of the starvation period. As with Lat B treatment, jasplakinolide pre-treatment had no effect on IRS1 tyrosine phosphorylation but was seen to mitigate insulin-induced disassociation of the IRS1/nexilin complex. Together, these results are consistent with the notion that insulin-elicited actin remodeling dynamically regulates IRS1– nexilin interactions.

**Figure 4:**
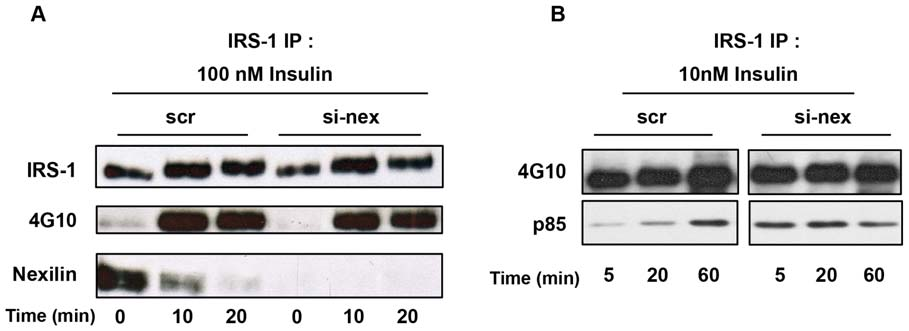
Silencing of nexilin enhances IRS1/PI3K assembly. L6 myotubes were transfected with either scrambled (scr) or nexilin specific siRNA (si-nex) oligos. Serum depleted cells were stimulated with 100 nM insulin *A*) or 10 nM *B*) for the indicated times. IRS1 was immunoprecipitated from cell lysates and complexes probed with either 4G10, nexilin or p85α PI3K abs as indicated.

**Figure 5:**
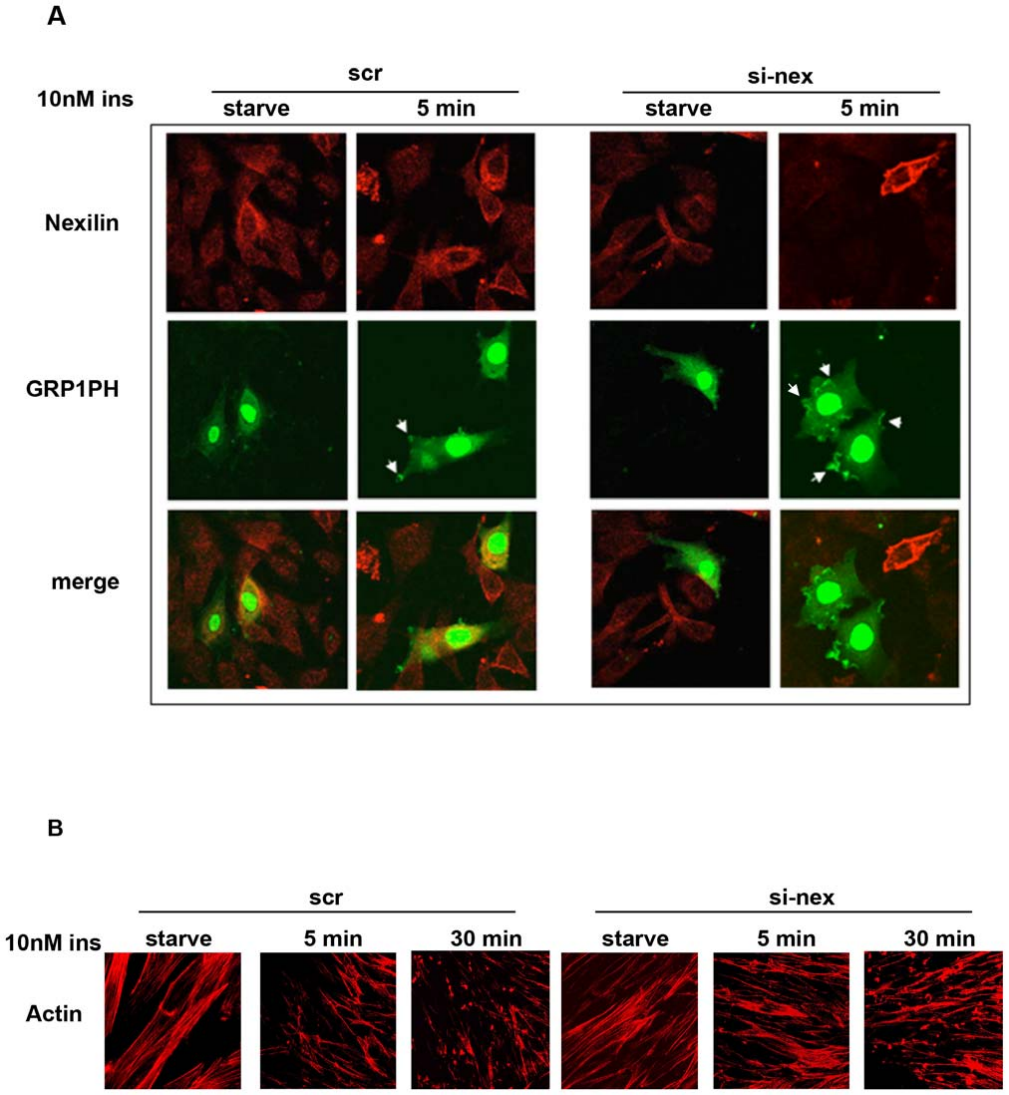
Silencing of nexilin enhances insulin-stimulated PIP_3_ production. *A*) L6 myoblasts were transfected with either scr or si-nex oligos together with GRP1-PH-GFP (GRP1PH) cDNA. Serum-starved cells were stimulated for 5 min with 10 nM insulin, fixed, permeabilized and incubated with anti-nexilin abs and Cy3-conjugated secondary abs (red). GFP was visualized using the appropriate filter. Arrows indicate regions of focal GRP1PH protein localization. *B*) L6 cells were transfected with either scr or si-nex oligos and left unstimulated or treated with 10 nM inulin for the indicated times. Cells were stained with rhodamine-phalloidin. Images were obtained on a Zeiss LSM510 laser scanning confocal microscope and manipulated using Canvas 9.04 (ACD Systems).

To study the functional requirement for nexilin in insulin-dependent signaling in skeletal muscle cells we began by assessing the effects of siRNA knockdown of nexilin (siNex) on the tyrosine phosphorylation status of IRS1 in response to insulin. As shown in Figure 4A disassembly of the IRS1/nexilin signaling complex correlated temporally with induction of IRS1 phosphorylation in response to insulin exposure, however, siRNA-mediated silencing of nexilin in L6 myotubes did not appear to have any discernible effects on the phosphotyrosine levels of IRS1 in cells incubated with maximal concentrations (100 nM) of insulin. We next sought to evaluate IRS1 tyrosine phosphorylation status under a submaximal dose (10 nM) of insulin. Interestingly, we found that silencing of nexilin under these conditions led to enhanced association of the p85/IRS1 signaling complex at earlier time points in the absence of any changes in IRS1 tyrosine phosphorylation (Fig. 4B). Thus our data would appear to indicate that the temporal release of IRS1 from nexilin/cytoskeletal scaffolds increases its coupling efficiency to downstream signalling intermediates. To test this idea, we assessed the effect of nexilin knockdown on PI3K activation and phosphatidylinositol-3,4,5-triphosphate (PIP_3_) production using single cell assays. To this end we made use of a fluorescent indicator consisting of green fluorescent protein (GFP)-tag fused to the pleckstrin homology (PH) domain of the general receptor for phosphoinositides-1 (GRP1). GRP1-PH-GFP has been shown to have a high affinity and strong selectivity for PIP_3_ (K_d_, ∼25 nM) [33] and as such we used it here as a surrogate marker of PI3K activity. L6 myoblasts were transiently transfected with siNex or siScr control oligos together with GRP1-PH-GFP and cells were subsequently serum-starved or stimulated 5 min with 10 nM insulin prior to being fixed and analyzed by confocal immunofluorescence analysis (Fig. 5). Under basal conditions, the GRP1-PH-GFP signal was prominently concentrated in the nucleus of L6 cells and faintly diffused throughout the cytosol as has previously been reported [33]. Upon exposure of siScr control cells to insulin, there was a small but notable concentration of GRP1-PH-GFP into distinct projections at the cell periphery, consistent with studies in L6 cells showing accumulation of PIP_3_ in actin remodeled structures at the dorsal surface of insulin-treated myoblasts [22] (Fig 5A, Left Panel, arrow heads). Significantly, in cells depleted of nexilin (as denoted by lack of nexilin staining), there was a pronounced increase in the intensity and size of GRP1-PH-GFP signals at peripheral membrane ruffles in insulin stimulated L6 cells, indicative of greater localized PIP_3_ production (Fig 5A, Right Panel). It is important to note that we did not observe any morphological changes in the actin cytoskeleton when nexilin was silenced under basal or insulin-stimulated conditions (Fig. 5B). Thus we propose that nexilin acts to constrain IRS1/PI3K complex formation and activation leading to PIP_3_ production at peripheral membranes.

**Figure 6:**
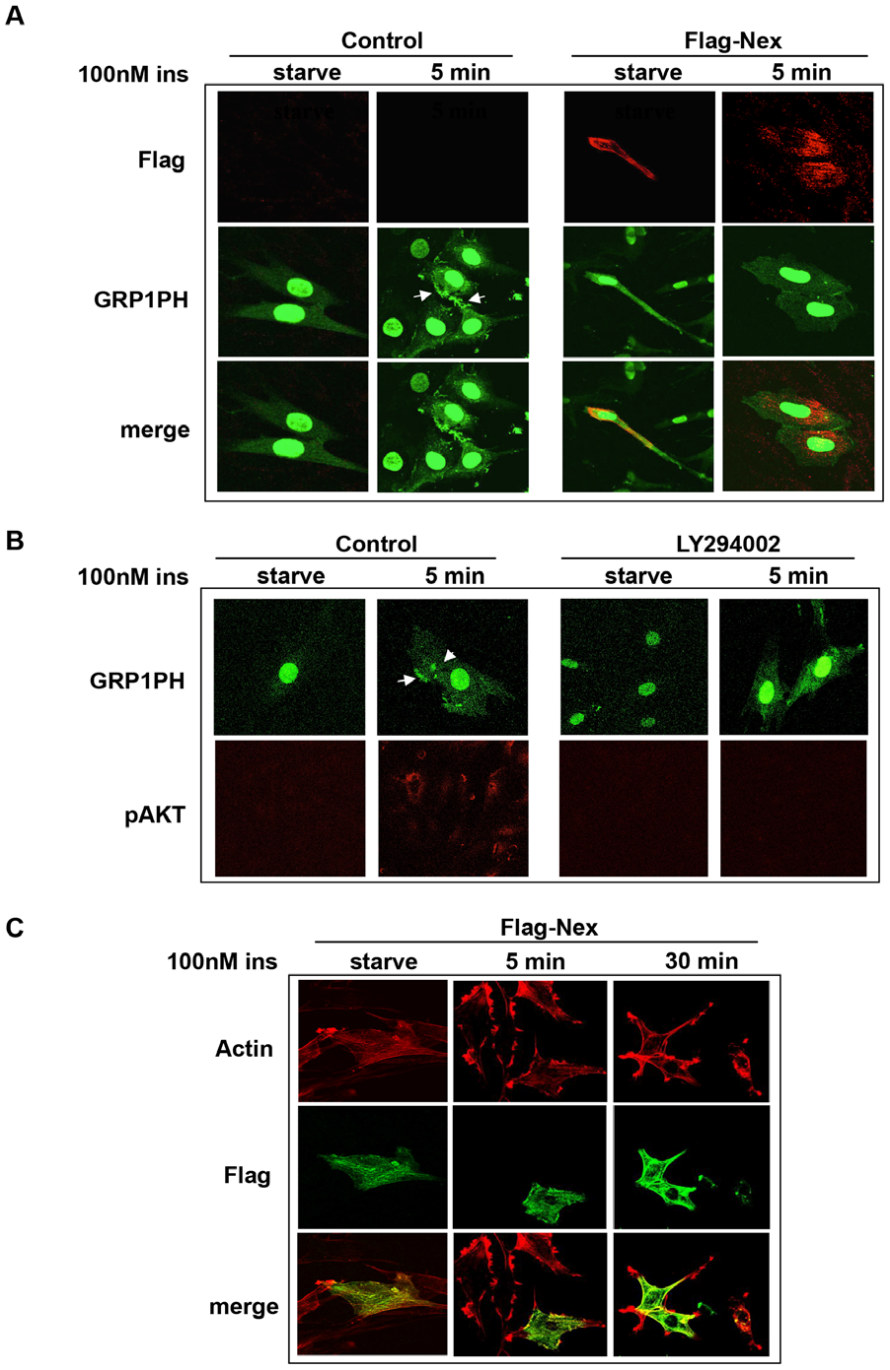
Overexpression of Flag-nexilin inhibits localized PI3K activation in L6 Cells. *A*) L6 myoblasts were transfected with Flag-nexilin or vector alone together with GRP1-PH-GFP cDNA. Following starvation, cells were stimulated with 100 nM insulin and then fixed, permeabilized and probed with anti-Flag antibodies followed by Cy3-conjugated donkey anti-mouse secondary abs (red). Cells were visualized for the presence of PIP3 accumulation in cell membranes using GRP1-PH-GFP. *B*) L6 cells were transfected with GRP1-PH-GFP and pretreated with Ly294002 (50 nM) prior to insulin stimulation and probed with anti-pAKT abs as in Figure 2. *C*) L6 myoblasts transfected with Flag-nexilin or vector alone were treated with 100 nM insulin for the indicated times and then probed with anti-Flag abs and Cy5-conjugated secondary abs (green) and rhodamine-phalloidin (red).

To test this idea further, we evaluated the effect of nexilin overexpression on insulin-stimulated PIP_3_ production in L6 cells. L6 myoblasts were transfected with either FLAG-nexilin or vector control together with GRP1-PH-GFP and left in serum-starved medium or stimulated with insulin , and subsequently subjected to confocal microscopy analysis (Fig 6). In control cells, insulin stimulation at a concentration of 100 nM evoked intense accumulation of PIP_3_ at membrane ruffles, whereas in Flag-nexilin positive L6 cells, this gain in GRP1-PH-GFP signals at the cell periphery was barely discernible (Fig. 6A). We noted that this inhibition of localized PIP_3_ production by Flag-nexilin was not associated with changes in insulin-induced formation of cortical actin bundles (Fig. 6C). Importantly, pre-treatment of L6 cells with the PI3K inhibitor LY294002 abolished the insulin-stimulated gain in GRP1-PH-GFP detection along the plasma membrane, confirming that mobilization of this reporter was dependent on PIP_3_ production (Fig. 6B).

**Figure 7:**
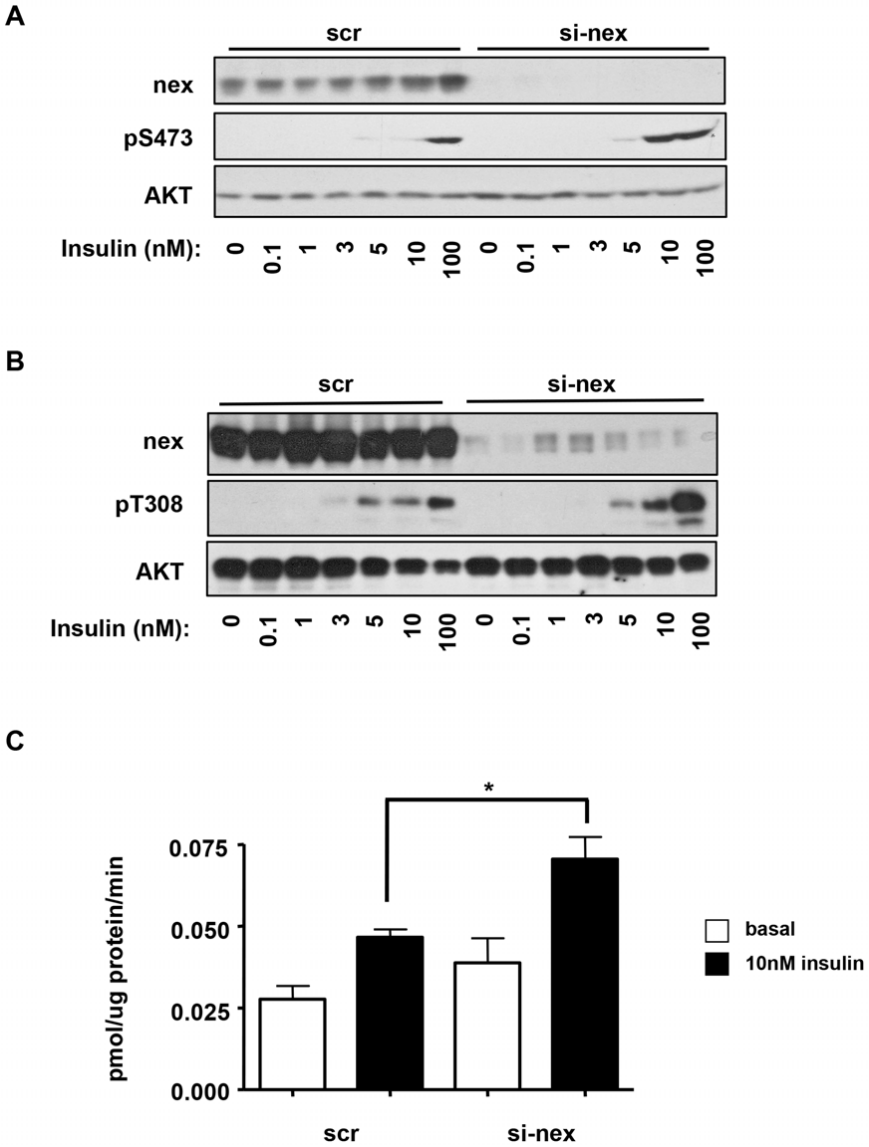
Silencing of nexilin enhances AKT activation and glucose uptake. *A,B*) L6 myotubes transfected with either control or si-nex oligos and stimulated with increasing concentrations of insulin for 5 minutes. Whole cell lysates were resolved by SDS/PAGE and immunoblotted with the indicated antibodies. *C*) Glucose uptake in L6 myotubes. Serum-starved cells were incubated with 10 nM insulin for 20 minutes prior to exposure to [^3^H] 2-deoxyglucose for 5 minutes. Data are means ± SE, n = 3 per group, one-way ANOVA (* *P*<0.05).

Given that Akt is a key mediator in the insulin-signaling pathway linking IRS1/PI3K activity to glucose uptake, we next tested the effect of nexilin knockdown on insulin-stimulated Akt phosphorylation. siRNA-treated L6 myotubes were incubated with a range of insulin concentrations for 5 min, and levels of Akt phosphorylation at serine 473 (S473) and threonine 308 (T308) were determined through immunoblot analysis. As shown in Figure 7A, siRNA-mediated depletion of nexilin in L6 myotubes led to sensitization of insulin-stimulated Akt S473 phosphorylation. Furthermore, analysis of T308 pAkT levels revealed that nexilin knockdown enhanced the robustness of the Akt response especially noticeable at 10 nM and 100 nM insulin doses (Fig. 7B). From these experiments it appears that nexilin might influence the quantitative characteristics of signals broadcast from the IRS/PI3K signalling node. Akt activation leads to the translocation of GLUT4 containing vesicles to the cell surface promoting the uptake of glucose into the cell. To determine the role of nexilin in GLUT4 transport, we measured glucose uptake in nexilin-depleted L6 myotubes. Consistent with our observation on Akt activation, nexilin knockdown significantly augmented insulin-stimulated 2-deoxyglucose uptake into siRNA-nexilin treated myotubes compared to control scr cells (Fig. 7C).

**Figure 8:**
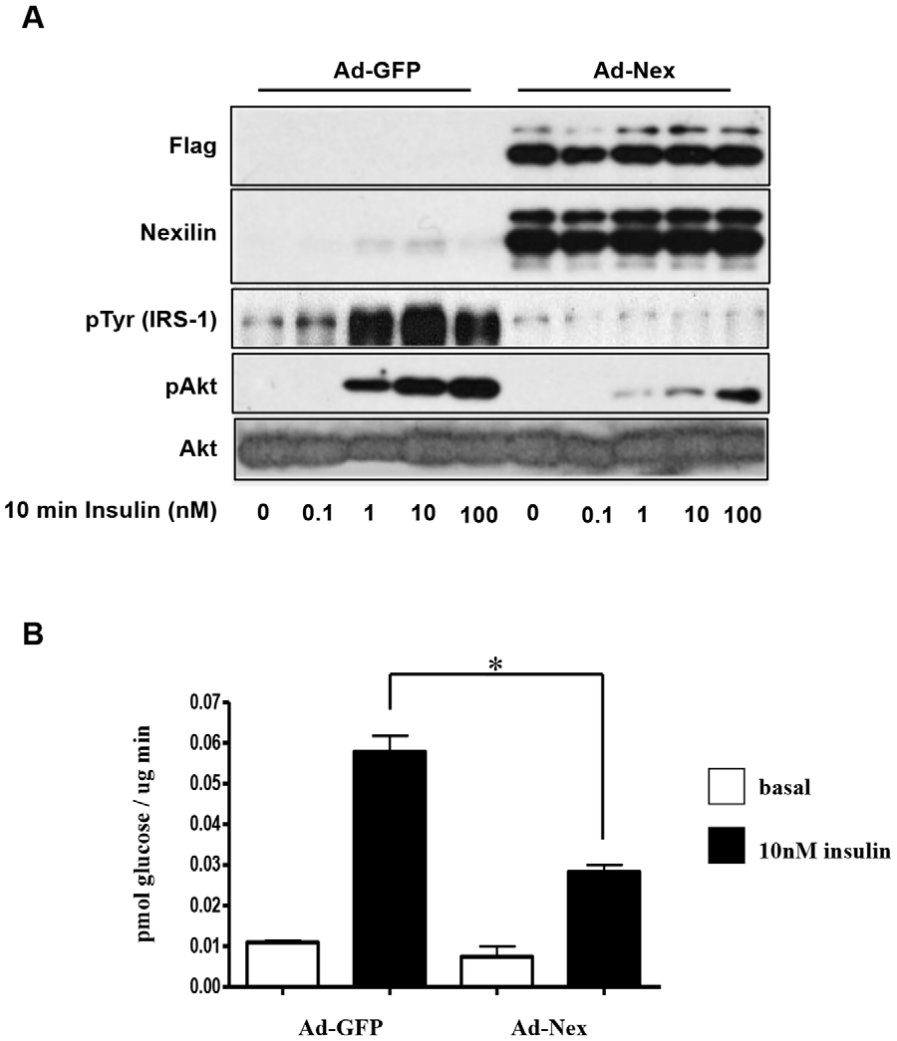
Overexpression of nexilin suppresses IRS1/PI3K signaling in 3T3-L1 adipocytes. *A*) 3T3-L1 adipocytes transduced with adenoviruses expressing FLAG-nexilin (ad-Nex) or GFP (Ad-GFP) were serum starved 72hrs post-infection and either left untreated or stimulated with increasing concentration of insulin. Cell lysates were subjected to Western blot analysis using the indicated antibodies. *B*) 3T3-L1 adipocytes transduced as in A) were starved or stimulated with 10 nM insulin and exposed to [^3^H] 2-deoxyglucose for 5 minutes. The reaction was ceased and the cells were harvested to assess the presence of [^3^H] within the cell through liquid scintillation counting. Data are means ± SE, n = 3 per group, one-way ANOVA (* *P*<0.001).

Given the abundance of nexilin in L6 cells, we chose to use 3T3-L1 adipocytes (3T3-L1) as a model system to investigate the effect of nexilin overexpression on insulin/IRS1 signaling as these cells express very low levels of nexilin. To this end, we generated adenoviruses expressing Flag-tagged nexilin (Ad-Nex) that efficiently transduced differentiated 3T3-L1s (Fig. 8A). Once infected with control Ad-GFP or Ad-Nex adenoviruses, 3T3-L1s were serum starved for at least 2 hours prior to treatment with a range of insulin doses. Our data revealed that nexilin overexpression caused a substantial reduction of insulin-stimulated Akt phosphorylation in cells treated with 1 nM and 10 nM insulin that coincided with significant inhibition of glucose uptake when compared to control Ad-GFP cells (Fig. 8A, B). There was no effect on glucose uptake when 100 nM of insulin was used (data not shown) which may reflect the transmission of insulin signals to GLUT4 through an IRS2-dependent mechanism. Surprisingly, we also observed that recombinant expression of nexilin in 3T3-L1s blocked insulin-induced tyrosine phosphorylation of IRS1. From these results we hypothesize that excess nexilin may lead to intracellular sequestration of IRS1, physically restricting its access to the activated insulin receptor.

In conclusion, we have identified nexilin as a novel negative regulator of IRS1-dependent signaling leading to glucose uptake. Furthermore, our findings raise the intriguing possibility that the selective binding of nexilin to IRS1 and not IRS2 may contribute to the differential specificity of IRS isoforms in the modulation of GLUT4 trafficking in skeletal muscle. In this study we demonstrate that nexilin plays a role in the insulin-dependent assembly and activation of the IRS1/PI3K/Akt signalosome. Under basal conditions, nexilin stably associates with IRS1 through the nexilin CC domain. Interestingly, hypertrophic cardiomyopathy-associated *NEXN* mutations in a cohort of Chinese patients were mapped to the ABD and CC domains of nexilin [24]. It was revealed that the ABD but not CC mutations abolished binding to α-actin in skeletal muscle. These data provide a molecular framework for the scaffolding function of nexilin in linking IRS-1 to the actin filament network which has been implicated in the spatial orchestration of IRS1 signaling events. Our findings suggest that nexilin limits access of IRS1 to PI3K activation-coupling without compromising insulin receptor substrate phosphorylation. Following insulin stimulation the interaction between nexilin and IRS1 is severed, presumably due to a process that is dependent on actin remodeling, as both Lat B and jasplakinolide treatment of L6 cells blocked efficient disassembly of the IRS1/nexilin complex in response to insulin. We show that insulin-elicited release of the inhibitory constraint exerted by nexilin enhances the efficiency of IRS1/PI3K interaction and PI-3, 4, 5-P_3_ production at localized sites which in turn impacts the sensitivity and magnitude of Akt activation. Given the localization of nexilin to the sarcomeric Z-disc, it would be of interest to assess whether it regulates contraction-stimulated glucose uptake in adult skeletal muscle. This study also opens doors to explore what role if any nexilin may play in the development of skeletal muscle insulin resistance. Moreover, it raises the possibility that loss of function mutations in the CC domain of nexilin that have been identified in patients with hypertrophic cardiomyopathy may be coupled to derangements in the IRS1/Akt signaling pathway.

## References

[1] Bjornholm M, Zierath JR (2005) Insulin signal transduction in human skeletal muscle: identifying the defects in Type II diabetes. Biochem Soc Trans 33: 354–357. 15787605 10.1042/BST0330354

[2] Stöckli1 J, Fazakerley DJ, James DE (2011) GLUT4 exocytosis. J Cell Sci 124: 4147–4159. 22247191 10.1242/jcs.097063 PMC3258103

[3] Hoffman NJ, Elmendorf JS (2011) Signaling, cytoskeletal and membrane mechanisms regulating GLUT4 exocytosis. Trends Endocrinol Metab 22: 110–116. 21216617 10.1016/j.tem.2010.12.001 PMC3049829

[4] Chiu TT, Jensen TE, Sylow L, Richter EA, Klip A (2011) Rac1 signalling towards GLUT4/glucose uptake in skeletal muscle. Cell Signal 23: 1546–1554. 21683139 10.1016/j.cellsig.2011.05.022

[5] Tsakiridis T, Vranic M, Klip A (1994) Disassembly of the actin network inhibits insulin-dependent stimulation of glucose transport and prevents recruitment of glucose transporters to the plasma membrane. J Biol Chem 269: 29934–29942. 7961991

[6] Khayat ZA, Tong P, Yaworsky K, Bloch RJ, Klip A (2000) Insulin-induced actin filament remodeling colocalizes actin with phosphatidylinositol 3-kinase and GLUT4 in L6 myotubes. J Cell Sci 113: 279–290. 10633079 10.1242/jcs.113.2.279

[7] Knight JB, Yamauchi K, Pessin JE (1995) Divergent insulin and platelet-derived growth factor regulation of focal adhesion kinase (pp125FAK) tyrosine phosphorylation, and rearrangement of actin stress fibers. J Biol Chem 270: 10199–10203. 7730324 10.1074/jbc.270.17.10199

[8] Kanzaki M, Watson RT, Khan AH, Pessin JE (2001) Insulin stimulates actin comet tails on intracellular GLUT4-containing compartments in differentiated 3T3L1 adipocytes. J Biol Chem 276: 49331–49336. 11606595 10.1074/jbc.M109657200

[9] Kanzaki M, Watson RT, Hou JC, Stamnes M, Saltiel AR, et al (2002) Small GTP-binding protein TC10 differentially regulates two distinct populations of filamentous actin in 3T3L1 adipocytes. Mol Biol Cell 13: 2334–2346. 12134073 10.1091/mbc.01-10-0490 PMC117317

[10] Sano H, Kane S, Sano E, Miinea CP, Asara JM, et al (2003) Insulin-stimulated phosphorylation of a Rab GTPase-activating protein regulates GLUT4 translocation. J Biol Chem 278: 14599–14602. 12637568 10.1074/jbc.C300063200

[11] Khayat TP, Huang ZA, Patel C, Ueyama U, Klip A (2001) Insulin-induced cortical actin remodeling promotes GLUT4 insertion at muscle cell membrane ruffles. J Clin Invest 108: 371–381. 11489930 10.1172/JCI12348 PMC209359

[12] Guilherme A, Emoto M, Buxton JM, Bose S, Sabini R, et al (2000) Perinuclear localization and insulin responsiveness of GLUT4 requires cytoskeletal integrity in 3T3-L1 adipocytes. J Biol Chem 275: 38151–38159. 10950952 10.1074/jbc.M003432200

[13] Omata W, Shibata H, Li L, Takata K, Kojima I (2000) Actin filaments play a critical role in insulin-induced exocytotic recruitment but not in endocytosis of GLUT4 in isolated rat adipocytes. Biochem J 346: 321–328. 10677349 PMC1220856

[14] Kanzaki M, Pessin JE (2001) Insulin-stimulated GLUT4 translocation in adipocytes is dependent upon cortical actin remodeling. J Biol Chem 276: 42436–42444. 11546823 10.1074/jbc.M108297200

[15] Emoto M, Langille SE, Czech MP (2001) A role for kinesin in insulin-stimulated glut4 glucose transporter translocation in 3T3-L1 adipocytes. J Biol Chem 276: 10677–10682. 11145966 10.1074/jbc.M010785200

[16] Eyster CA, Duggins QS, Olson AL (2005) Expression of Constitutively Active Akt/Protein Kinase B Signals GLUT4 Translocation in the Absence of an Intact Actin Cytoskeleton. J Biol Chem 280: 17978–17985. 15738003 10.1074/jbc.M409806200

[17] GInoue G, Cheatham B, Emkey R, Kahn CR (1998) Dynamics of insulin signaling in 3T3-L1 adipocytes. Differential compartmentalization and trafficking of insulin receptor substrate (IRS)-1 and IRS-2. J Biol Chem 273: 11548–11555. 9565570 10.1074/jbc.273.19.11548

[18] Kriauciunas KM, Myers MGJ, Kahn CR (2000) Cellular compartmentalization in insulin action: altered signaling by a lipid-modified IRS-1. Mol Cell Biol 20: 6849–6859. 10958681 10.1128/mcb.20.18.6849-6859.2000 PMC86221

[19] Anai M, Ono H, Funaki M, Fukushima Y, Inukai K, et al (1998) Different subcellular distribution and regulation of expression of insulin receptor substrate (IRS)-3 from those of IRS-1 and IRS-2. J Biol Chem 273: 29686–29692. 9792680 10.1074/jbc.273.45.29686

[20] Clark SF, Martin S, Carozzi AJ, Hill MM, James DE (1998) Intracellular localization of phosphatidylinositide 3-kinase and insulin receptor substrate-1 in adipocytes: potential involvement of a membrane skeleton. J Cell Biol 140: 1211–1225. 9490733 10.1083/jcb.140.5.1211 PMC2132698

[21] Clark SF, Molero JC, James DE (2000) Release of insulin receptor substrate proteins from an intracellular complex coincides with the development of insulin resistance. J Biol Chem 275: 3819–3826. 10660532 10.1074/jbc.275.6.3819

[22] Patel N, Rudich A, Khayat ZA, Garg R, Klip A (2003) Intracellular segregation of phosphatidylinositol-3,4,5-trisphosphate by insulin-dependent actin remodeling in L6 skeletal muscle cells. Mol Cell Biol 23: 4611–4626. 12808101 10.1128/MCB.23.13.4611-4626.2003 PMC164845

[23] Hassel D, Dahme T, Erdmann J, Meder B, Huge A, et al (2009) Nexilin mutations destabilize cardiac Z-disks and lead to dilated cardiomyopathy. Nat Med 15: 1281–1288. 19881492 10.1038/nm.2037

[24] Frank D, Frey N (2011) Cardiac Z-disc Signaling Network. J Biol Chem 286: 9897–9904. 21257757 10.1074/jbc.R110.174268 PMC3060542

[25] Wang H, Li Z, Wang J, Sun K, Cui Q, et al (2010) Mutations in NEXN, a Z-disc gene, are associated with hypertrophic cardiomyopathy. Am J Hum Genet 87: 687–693. 20970104 10.1016/j.ajhg.2010.10.002 PMC2978958

[26] Waardenberg AJ, Bernardo BC, Ng DC, Shepherd PR, Cemerlang N, et al (2011) Phosphoinositide 3-Kinase (PI3K(p110α)) Directly Regulates Key Components of the Z-disc and Cardiac Structure. J Biol Chem 286: 30837–30846. 21757757 10.1074/jbc.M111.271684 PMC3162444

[27] Shimizu I, Minamino T, Toko H, Okada S, Ikeda H, et al (2010) Excessive cardiac insulin signaling exacerbates systolic dysfunction induced by pressure overload in rodents. J Clin Invest 120: 1506–1514. 20407209 10.1172/JCI40096 PMC2860916

[28] Samuelsson AM, Bollano E, Mobini R, Larsson BM, Omerovic E, et al (2006) Hyperinsulinemia: effect on cardiac mass/function, angiotensin II receptor expression, and insulin signaling pathways. Am J Physiol Heart Circ Physiol 291: 787–796. 10.1152/ajpheart.00974.2005 16565309

[29] Thirone AC, Huang C, Klip A (2006) Tissue-specific roles of IRS proteins in insulin signaling and glucose transport. Trends Endocrinol Metab 17: 72–78. 16458527 10.1016/j.tem.2006.01.005

[30] Huang C, Thirone AC, Huang X, Klip A (2005) Differential contribution of insulin receptor substrates 1 versus 2 to insulin signaling and glucose uptake in L6 myotubes. J Biol Chem 280: 19426–19435. 15764603 10.1074/jbc.M412317200

[31] Yamauchi T, Tobe K, Tamemoto H, Ueki K, Kaburagi Y, et al (1996) Insulin signalling and insulin actions in the muscles and livers of insulin-resistant, insulin receptor substrate 1-deficient mice. Mol Cell Biol 16: 3074–3084. 8649419 10.1128/mcb.16.6.3074 PMC231302

[32] Higaki Y, Wojtaszewski JF, Hirshman MF, Withers DJ, Towery H, et al (1999) Insulin receptor substrate-2 is not necessary for insulin- and exercise-stimulated glucose transport in skeletal muscle. J Biol Chem 274: 20791–20795. 10409618 10.1074/jbc.274.30.20791

[33] Oatey PB, Venkateswarlu K, Williams AG, Fletcher LM, Foulstone EJ, et al (1999) Confocal imaging of the subcellular distribution of phosphatidylinositol 3,4,5-trisphosphate in insulinand PDGF-stimulated 3T3-L1 adipocytes. Biochem J 344: 511–518. 10567235 PMC1220670

